# Not stealing from the treasure chest (or just a bit): Analyses on plant derived writing supports and non-invasive DNA sampling

**DOI:** 10.1371/journal.pone.0198513

**Published:** 2018-06-06

**Authors:** Anna Schulz, Silke Lautner, Jörg Fromm, Markus Fischer

**Affiliations:** 1 Hamburg School of Food Science, University of Hamburg, Hamburg, Germany; 2 Centre for Wood Science, University of Hamburg, Hamburg, Germany; University of Helsinki, FINLAND

## Abstract

Written communication plays a crucial role in the history of modern civilizations as manuscripts do not only exist contemporarily, but are passed on to subsequent generations. Besides a document’s content, information is stored in the materials used for its production. Analyses of the composition allow, for example, identifying the biological origins of materials, dating, and help to understand degradation patterns. A combination of microscopic and DNA approaches was applied in order to analyze various plant derived writing sheets. Given their diversity and abundance in museum collections, plant based writing supports are yet an underexplored target for DNA studies. DNA retrieval of paper is low compared to raw paper plant material, which is likely due to the loss of organic components during paper production. Optimizing DNA extraction for each respective material drastically increased DNA recovery. Finally, we present a non-invasive DNA sampling method that utilizes nylon membranes, commonly used for bacterial DNA sampling and that is applicable to delicate material. Although bacterial infestation was visible on one sample, as indicated by scanning electron microscopy, endogenous DNA was retrieved. The results presented here are promising as they extend the scope of sources for DNA analyses by demonstrating that DNA molecules can be retrieved from a variety of plant derived writing supports. In future, such analyses can help to explore the biological diversity not only of plants and of additives utilized for producing writing supports, but also of the plenty products made from paper.

## Introduction

Throughout history, versatile materials were utilized as writing support, starting with Paleolithic cave rocks to Bronze Age clay tablets to Iron Age parchment, modern paper, and novel stone paper. Besides a document’s content, information is stored in the materials used for its production. Analyses of the composition allow, for example, identification of materials [1–7], dating [8, 9], and help to understand degradation patterns [6, 10, 11]. Given their abundance in museum collections and the diversity of writing supports made of plant material, such as papyrus scrolls, wood tablets, and paper, with one exception [12], plant derived manuscripts are an underexplored target for DNA analyses. In theory, DNA molecules can be obtained from any organic material such as bone [13, 14], eggshell [15], wood [16, 17], as well as from processed material, for example food [18, 19], clothing [20–22], and parchment [1–4, 23]. The survival and preservation of DNA molecules is not only influenced by the age of a sample, but to a higher degree by the organic source [24–26] and the environment [27, 28], with arid conditions and low temperature fluctuations favoring long time DNA survival. For processed material, the manufacturing process will additionally influence molecular preservation [7, 21, 23]. Preparing manuscript sheets from a variety of organic materials involves physical force (cutting and pressing) as well as thermal and chemical treatment (soaking or boiling in alkaline solutions). Sheets were often bleached, tanned or coated with animal or plant derived sizes for improved ink absorption [29, 30]. Because the production of paper requires mainly cellulosic material and water, paper can be and has been manufactured not only from fresh bark and wood, but also from textile waste, such as linen, hemp, and cotton [30].

Another limitation for DNA analyses is the sensitivity of the applied method, especially during the isolation of DNA molecules. Protocols do exist for the isolation of DNA molecules from fresh and herbarium leaf samples [24, 31, 32], but their efficiency to retrieve DNA from the diverse plant derived writing supports has not been tested. Palm leaves, bark, and pith- the raw material for papyrus- are lignified and are probably more difficult to dissolve in order to release DNA molecules. Concerning cultural heritage, research is restricted by the need of material integrity. DNA analyses are usually invasive, as they require removal of material. In order to study precious samples, such as museum collections, rare or small specimens, it is necessary to develop non-destructive sampling techniques. Non-destructive sampling can be thought of removing sampling material while keeping the material intact. For example, incubating material in lysis buffer was successfully performed for sampling DNA from bones [33] and insects [34, 35], while scraping was applied for sampling residues in ceramics found in a shipwreck [36]. Non-invasive or minimally invasive sampling will remove sampling material without penetrating the surface of a material, for example by using swabs. Recent studies have demonstrated the ability of analyzing parchment [37] and herbarium specimens [31] by applying eraser sampling, although it has been noted, that this method is not applicable to delicate samples, as they become easily damaged [31]. The usage of binding membranes offers another non-invasive sampling method. Nitrocellulose and nylon membranes have been applied for bacterial and fungal sampling from the surface of paintings [38], manuscripts [39], and photographs [40] so far, but have not been tested in their efficiency to bind endogenous DNA.

Here, we have analyzed samples of Asian paper, papyrus, and a historic palm leaf manuscript by using different extraction protocols. In order to monitor the effect of paper production on DNA survival, material of paper mulberry (*Broussonetia papyrifera*) was analyzed at different manufacturing steps (raw bark, cooked bark, modern unbleached and bleached paper sheets, and an early 20^th^ century unbleached paper sheet). We successfully tested a non-invasive DNA sampling technique that is applicable to delicate material, such as Asian paper. We identified the biological origin of the 18^th^ century palm leaf manuscript by independent DNA and microscopy approaches. In particular, light microscopy and scanning electron microscopy (SEM) were applied, two independent and widespread tools to analyze plant structure. Light microscopy provides a rapid method and is used to study cells of the order of magnitude 1 μm to several mm. For the analyses of leaf samples it provides information on tissue patterns, cell types, and for species identification [41]. In contrast to optical microscopy, SEM reaches much higher magnification and has been used over the years for looking at numerous aspects of plant structure, for example pollen grains [42], plant cell walls [43], and leaf surfaces [44].

## Results and discussion

### Effect of paper production

In brief, the manufacture of Asian bark paper involves watering or cooking of raw bark in the presence of alkaline additives. The fibers then are washed, separated manually or mechanically by beating, and mixed with water. The resulting pulp is poured on a casting mold and left to dry [45]. Based on a total of 11 different extraction protocols the average DNA content (given as ng DNA per mg of input tissue) was calculated for bark paper at different production steps (raw bark, cooked, bark, unbleached paper, bleached paper). Major differences in DNA content were observed between raw bark material and processed paper. Cooked bark yielded more DNA than raw bark tissue (on average 26.3 and 10.6 ng/mg, respectively, t test p-value = 0.001, see Tables 1 and 2), which can be most likely attributed to incomplete tissue disintegration during lysis. Dry bark is extremely hard and dense, and dissolved hardly after 24 h incubation in lysis buffer. Because of cooking, the release of DNA molecules might be facilitated, while raw material needs prolonged incubation in order to disrupt the tissue matrix. Homogenizing raw bark material in lysis buffer for 72 h led to a significant increase in the DNA output (69.5 ng/mg, t test p-value = 0.05). Significant differences in PCR success were observed between raw and cooked material with respect to amplicon length. Cooked bark showed lower amplification success for longer fragments (i.e. 791 base pairs (bp) and 500 bp, t test p-values = 0.006). Depending on the plant source, cooking of raw bark material can take up to several hours [45] and heat induced fragmentation of DNA molecules [46] during this step of paper preparation maybe a reason.

**Table 1:** Average DNA yield and amplification success of *Broussonetia papyrifera* at different paper production steps. PCR success is given as the number of PCR products (visualized by gel electrophoresis) for different amplicon lengths (791, 500, and 120bp). Standard deviation for DNA yields is given in parentheses.

	DNA yield (ng/mg)	PCR success rate (%)
Bark, raw	10.58 (10.04)^a^	791/16 (42), 500/20 (61), 120/24 (73)
	23.28 (8.88)^b^	
	31.25 (3.89)^c^	
	69.5 (3.54)^d^	
Bark, cooked	26.29 (17.03)^a^	791/9 (27), 500/13 (39), 120/22 (67)
Paper, modern, unbleached	3.88 (1.75)^a^	791/10 (30), 500/12 (36), 120 15 (45)
	5.63 (2.3)^e^	120/4 (67)^e^
Paper, modern, bleached	2.16 (1.8)^a^	791/2 (6), 500/7 (21), 120/9 (27)
Paper, *c*. 100 yrs old, unbleached	5.73 (3.57)^e^	120/6 (100)^e^

^a^ DNA yield after 24 h of incubation in different lysis buffers

^b^ DNA yield after 24 h of incubation in CTAB + SDS lysis buffer.

^c^ DNA yield after 48 h of incubation in CTAB + SDS lysis buffer.

^d^ DNA yield after 72 h of incubation in CTAB + SDS lysis buffer.

^e^ DNA yield and PCR amplification success after extraction with PeqGOLD Plant DNA Mini kit and EDTA + NaCl + SDS.

**Table 2:** Results of t tests for paper manufacturing experiments. p-values< 0.05 are highlighted. n = sample number.

Test	DF	T	p-value
Differences in DNA yield:			
**Raw bark, cooked bark (n = 2)**^a^	10	**-4.634**	**0.001**
**Cooked bark, modern unbleached paper (n = 2)**	10	**4.381**	**0.001**
**Modern unbleached paper, modern bleached paper (n = 2)**	10	**3.494**	**0.006**
Modern unbleached paper, historic unbleached paper (n = 2)^b^	1	-0.111	0.930
Influence of incubation time on DNA yield of raw bark:			
24 h, 48 h (n = 2)	1	-0.883	0.540
**24 h, 72 h (n = 2)**	1	**-12.228**	**0.050**
Differences amplification success: 791 bp:			
**Raw bark, cooked bark (n = 2)**	32	**2.935**	**0.006**
Cooked bark, unbleached paper (n = 2)	32	1.000	0.325
**Unbleached paper, bleached paper (n = 2)**	32	**3.200**	**0.003**
Differences in amplification success: 500 bp:			
**Raw bark, cooked bark (n = 2)**	32	**2.935**	**0.006**
Cooked bark, unbleached paper (n = 2)	32	-1.000	0.325
**Unbleached paper, bleached paper (n = 2)**	32	**2.390**	**0.023**
Differences in amplification success: 120 bp:			
Raw bark, cooked bark (n = 2)	32	1.437	0.160
**Cooked bark, unbleached paper (n = 2)**	32	**2.935**	**0.006**
**Unbleached paper, bleached paper (n = 2)**	32	**2.667**	**0.012**

^a^ DNA yield after 24 h of incubation in lysis buffer.

^b^ DNA yield when comparing the same extraction methods.

To test whether DNA degradation had an effect on the differences in amplification success, DNA sequence lengths were measured on a Bioanalyzer. No shifts in fragment lengths were observed between raw and cooked bark as well as modern, unbleached paper, while measurements of bleached paper failed to produce a measurement. Rather than fragmentation, it appears that alkaline chemicals added during cooking inhibit PCR amplification.

**Fig 1:**
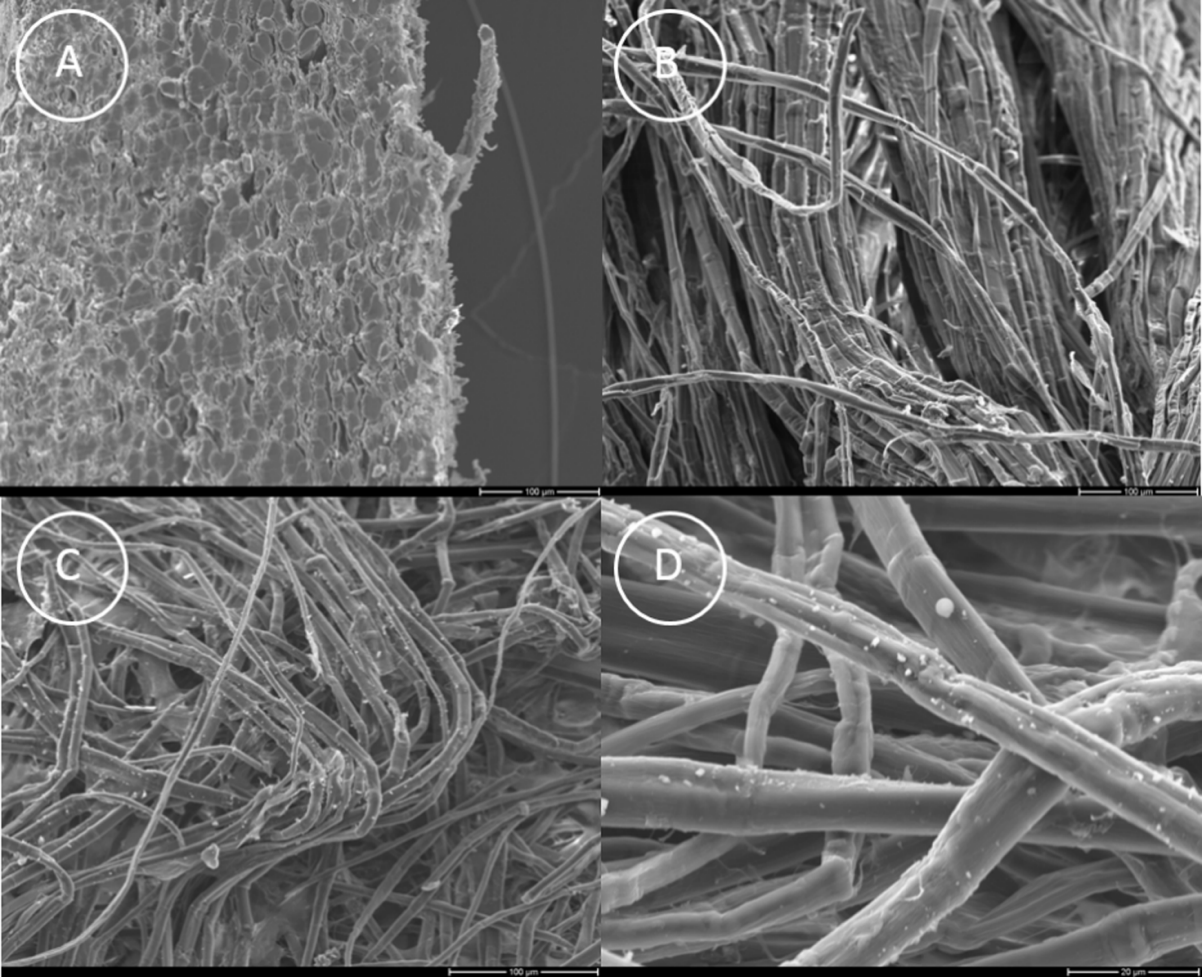
SEM images from *Broussonetia papyrifera*. (A) Raw bark material. Overview of the transverse section. Within the various cell types (parenchyma cells, fibers, sieve elements, companion cells) fibers had the highest content (> 50%). (B) Cooked bark material. Numerous bends are evident and the fibers appear more or less wrinkled. (C). Unbleached paper showing several mm long fibers. (D) Detailed view on the fiber surface showing several bends and variations in fiber thickness from 3 to 15 μm.

DNA yield was significantly lower in paper compared to cooked bark (t test p-value = 0.001), which is most likely attributed to a loss of organic compounds during pulping. Fibers consist of cellulose, lignin and hemicelluloses. A high content of cellulose is the key determinant of paper strength. In order to obtain cellulose, chemical (using alkaline solutions) and/or mechanical (by beating or grinding) pulping is performed, which results in the dissolution of nearly all lignin and more than half of the hemicellulose content from the fibers, whereas cellulose is partly degraded. During the removal of lignin and hemicellulose, the pore volume increases and the fiber surface becomes more open (Fig 1) [47]. Lignin is hydrophobic and is associated with material durability [48], protection of cellulose and hemicellulose from degradation, protection of cell walls [49], and pathogen resistance [50], and may be linked also to the good molecular preservation of the historic palm leaf (see below) and other lignified, ancient tissues [16, 51–53].

Within paper, DNA content was highest in unbleached samples (3.9 ng/mg for modern paper and 5.7 ng/mg for the historic specimen). Due to limited sample amount, DNA extraction was performed only twice for the historic paper specimen including the protocol that obtained the highest DNA yield for paper (EDTA+ NaCl+ SDS lysis). The DNA yield of the historic paper specimen did not differ significantly to modern, unbleached paper (5.6 ng/mg) when comparing the same extraction methods only (t test p-value = 0.930). Although a bias due to limited analyses cannot be rejected for the historic paper sample, one should keep in mind that historic and modern paper production differ in their level of chemical input. Traditionally, water filtered through ashes is used for cooking bark material and is nowadays replaced by adding alkaline chemicals, such as potassium carbonate.

Bleaching had a negative effect on both DNA recovery (2.2 ng/mg compared to 3.9 ng/mg, t test p-value = 0.006) and overall PCR amplification success (6–27% compared to 30–45% for unbleached modern paper, t test p-values< 0.05, Tables 1 and 2). The paper sheet analyzed here was treated with chemical bleach, which is likely to be more detrimental to DNA survival than traditional bleaching by sun or snow.

DNA fragments of the historic paper sample ranged around 80 base pairs (bp). Given the moderate age of this sample (early 20^th^ century), this finding suggests that DNA fragmentation occurs relatively fast. This was also observed in the palm leaf manuscript analyzed here (*c*. 140 bp), and in accordance to previous studies on historic specimens of younger date [24, 54, 55].

### DNA recovery of plant derived manuscripts using different lysis buffers

A total of eleven lysis buffers and varying incubation times were tested in order to identify the best method for each respective plant derived writing support (modern papyrus, modern unbleached paper, historic palm leaf manuscript) and other plant material (fresh soft leaves and raw bark). Within samples of the same material (i.e. raw bark, leaves, modern, unbleached paper), significant differences in the DNA yield were only observed between fresh, alcohol preserved leaves (t test p-value = 0.002, see Table 3). The leaves were sampled and analyzed at the same time, and were preserved and stored under the same conditions. It therefore seems unlikely that these factors had an effect on the results.

**Table 3:** T test results of extraction comparisons. p-values< 0.05 are highlighted. n = sample size.

Test	DF	T	p-value
Differences in DNA yield within the same material source:			
**Fresh alcohol preserved leaves (n = 2)**	32	**-3.285**	**0.002**
Raw bark (n = 3)^a^	9	-0.507 (±0.79)	0.685 (±0.43)
Unbleached modern paper (n = 3)^a^	9	-0.044 (±0.32)	0.822 (±0.11)
Differences in DNA yield between buffers:			
**Fresh alcohol preserved leaves: CTAB, EDTA (n = 2)**^b^	29	**3.395**	**0.002**
**Raw bark: CTAB, EDTA (n = 3)**^b^	29	**4.469**	**0.000**
**Modern unbleached paper: CTAB, EDTA (n = 3)**^b^	29	**-3.221**	**0.003**
Papyrus: CTAB, EDTA (n = 1)^b^	29	-1.246	0.223
**Palm leaf: CTAB, EDTA (n = 1)**^b^	29	**-4.394**	**0.000**
Fresh alcohol preserved leaves: Plant DNA kit, CTAB (n = 2)^c^	5	-2.075	0.093
Raw bark: Plant DNA kit, CTAB (n = 3)^c^	5	-1.902	0.116
**Modern unbleached paper: Plant DNA kit, EDTA (n = 3)**^c^	5	**-7.317**	**0.001**
**Papyrus: Plant DNA kit, EDTA (n = 1)**^c^	5	**-5.347**	**0.003**
**Palm leaf: Plant DNA kit, EDTA (n = 1)**^c^	5	**-8.120**	**0.000**
Differences in amplification success between buffers:			
**114/120 bp: CTAB, EDTA**^d^	134	**2.905**	**0.004**
**500 bp: CTAB, EDTA**	134	**4.968**	**0.000**
791 bp: CTAB, EDTA	134	1.392	0.166
**Fresh alcohol preserved leaves: CTAB, EDTA (n = 2)**	89	**3.145**	**0.002**
**Raw bark: CTAB, EDTA (n = 3)**	134	**2.023**	**0.045**
**Modern unbleached paper: CTAB, EDTA (n = 3)**	134	**4.540**	**0.000**
Papyrus: CTAB, EDTA (n = 1)	44	1.773	0.785
Influence of incubation time on DNA yield:			
**Papyrus, palm leaf, modern unbleached paper: 24 h, 6 h (n = 5)**	17	**3.571**	**0.002**
**Papyrus, palm leaf, modern unbleached paper: 24 h, 48 h (n = 5)**	17	**3.184**	**0.005**
**Papyrus, palm leaf, modern unbleached paper: 24 h, 72 h (n = 5)**	17	**3.646**	**0.002**
Differences in amplification success using different incubation times:			
Papyrus, paper, palm leaf: 24 h, 6 h	38	1.707	0.096
Papyrus, paper, palm leaf: 24 h, 48 h	38	0.902	0.373
**Papyrus, paper, palm leaf: 24 h, 72 h**	38	**2.731**	**0.010**

^a^ average values when comparing the same material input within the same material.

^b^ comparison between all tested CTAB and EDTA based lysis buffers.

^c^ comparison between PeqGOLD Plant DNA kit with the best respective lysis buffer.

^d^ excluding the historic palm leaf sample.

CTAB-based lysis buffers, commonly used for materials rich in polyphenols and polysaccharides [56], yielded the highest DNA amounts for fresh leaves and bark material (199.9 ng/mg and 24.4 ng/mg, respectively, Table 4). Buffers containing mainly EDTA, a chelating agent that prevents DNA degradation by nucleases and is used for decalcification during DNA extraction from bones, yielded highest DNA amounts for paper and the palm leaf. For papyrus no significant difference was observed between CTAB or EDTA based lysis buffers (6.01 ng/mg and 7.5 ng/mg, respectively, t test p-value = 0.223), but a significant increase in DNA yield was observed in comparison to the PeqGOLD Plant DNA kit (t test p-value = 0.003, Table 3).

**Table 4:** DNA yield using different extraction lysis buffers and amplification success. n = sample size. Each extraction method was tested six times on each material. PCR and qPCR were conducted in triplicate for each sample. Standard deviation is given in parentheses. PCR success is given as the number of PCR products (visualized by gel electrophoresis) for different amplicon lengths (791 bp, 500 bp, and 114/ 120 bp). n.a. = not analyzed.

Method		unbleached, modern paper (n = 3)	modern papyrus (n = 1)	18^th^ century palm leaf (n = 1)	fresh leaf (n = 2)	raw bark (n = 3)
Plant DNA Mini kit, PeqGold	ng/mg	2.7 (1.27)	1.28 (0.13)	11.02 (1.49)	30.31 (12.79)	10.47 (10.85)
	OD260/280^a^	1.50 (0.12)	1.65 (0.05)	1.80 (0.12)	1.78 (0.05)	1.90 (0.22)
	PCR (%)	791/4 (44), 500/5 (56), 120/6 (67)	791/3 (100), 500/3 (100), 114/3 (100)	114/3 (100)	791/6 (100), 500/6 (100), 120/6 (100)	791/7 (78), 500/6 (67), 120/9 (100)
CTAB	ng/mg	0.98 (0.48)	6.55 (7.14)	66.38 (50.07)	72.67 (29.28)	12.27 (9.05)
	OD260/280	1.82 (0.17)	1.79 (0.20)	1.91 (0.07)	1.90 (0.06)	1.99 (0.05)
	PCR (%)	791/4 (44), 500/5 (56), 120/5 (56)	791/2 (67), 500/2 (67), 114/3 (100)	114/3 (100)	791/6 (100), 500/6 (100), 120/6 (100)	791/6 (67), 500/6 (67), 120/4 (44)
CTAB + SDS	ng/mg	2.49 (0.76)	5.27 (5.30)	56.40 (51.11)	101.82 (65.96)	24.37 (9.89)
	OD260/280	1.61 (0.35)	1.64 (0.13)	2.08 (0.20)	1.94 (0.04)	2.05 (0.13)
	PCR (%)	791/4 (44), 500/6 (67), 120/6 (67)	791/2 (67), 500/2 (67), 114/3 (100)	114/3 (100)	791/6 (100), 500/6 (100), 120/6 (100)	791/5 (56), 500/7 (78), 120/7 (78)
CTAB + DTT + SDS	ng/mg	2.83 (1.66)	6.58 (4.30)	52.41 (25.27)	199.90 (206.63)	12.13 (7.59)
	OD260/280	1.85 (0.16)	1.65 (0.05)	1.79 (0.36)	1.89 (0.11)	1.82 (0.07)
	PCR (%)	791/4 (44), 500/3 (33), 120/6 (67)	791/1 (33), 500/2 (67), 114/3 (100)	114/3 (100)	791/3 (50), 500/6 (100), 120/6 (100)	791/6 (67), 500/7 (78), 120/8 (89)
CTAB + SDS + column	ng/mg	3.36 (1.34)	6.28 (2.88)	16.71 (6.64)	145.05 (162.57)	6.9 (3.25)
	OD260/280	1.47 (0.25)	1.75 (0.07)	1.78 (0.20)	2.00 (0.06)	1.56 (0.42)
	PCR (%)	791/3 (33), 500/3 (33), 120/4 (44)	791/1 (33), 500/2 (67), 114/3 (100)	114/3 (100)	791/5 (83), 500/4 (67), 120/6 (100)	791/6 (67), 500/6 (67), 120/8 (89)
CTAB + BME + DTT + SDS	ng/mg	3.2 (1.63)	5.39 (2.25)	52.00 (14.79)	75.95 (100.27)	7.93 (3.99)
	OD260/280	1.85 (0.43)	1.72 (0.05)	1.78 (0.15)	1.61 (0.21)	1.37 (0.43)
	PCR (%)	791/3 (33), 500/3 (33), 120/3 (33)	791/1 (33), 500/2 (67), 114/3 (100)	114/3(100)	791/5 (83), 500/4 (67), 120/6 (100)	791/2 (22), 500/5 (56), 120/6 (67)
sum CTAB^b^	ng/mg	2.57 (1.44)	6.01 (4.34)	48.78 (35.88)	119.08 (127.94)	12.72 (9.09)
	PCR (%)	791/18 (40), 500/20 (44), 120/24 (53)	791/7 (47), 500/10 (67), 114/15 (100)	114/15 (100)	791/25 (83), 500/26 (87), 120/30 (100)	791/25 (56), 500/31 (69), 120/33 (73)
EDTA + DTT + SDS + ProtK	ng/mg	4.11 (3.26)	3.02 (1.82)	104. 97 (31.91)	5.77 (3.52)	4.53 (2.78)
	OD260/280	0.55 (0.21)	2.03 (0.53)	2.40 (0.49)	2.24 (0.31)	2.03 (0.58)
	PCR (%)	791/1 (11), 500/3 (33), 120/4 (44)	791/2 (67), 500/1 (33), 114/2 (67)	114/3 (100)	791/4 (67), 500/4 (67), 120/5 (83)	791/2 (22), 500/5 (56), 120/5 (56)
EDTA + NaCl + DTT + SDS	ng/mg	3.77 (1.15)	4.67 (2.01)	41.79 (9.84)	29.85 (11.25)	3.10 (1.59)
	OD260/280	1.71 (0.33)	1.39 (0.09)	1.72 (0.15)	1.89 (0.13)	2.01 (0.08)
	PCR (%)	791/3 (33), 500/3 (33), 120/6 (67)	791/2 (67), 500/1 (33), 114/2 (67)	114/3 (100)	791/5 (83), 500/4 (67), 120/6 (100)	791/9 (100), 500/5 (56), 120/7 (78)
EDTA + NaCl + SDS	ng/mg	8.66 (2.23)	6.94 (2.51)	83.48 (27.63)	63.14 (43.79)	8.2 (4.07)
	OD260/280	1.90 (0.15)	1.77 (0.19)	2.38 (0.33)	2.55 (0.37)	1.23 (0.15)
	PCR (%)	791/3 (33), 500/3 (33), 120/5 (56)	791/2 (67), 500/2 (67), 114/3 (100)	114/3 (100)	791/6 (100), 500/4 (67), 120/6 (100)	791/5 (56), 500/6 (67), 120/3 (33)
EDTA + DTT + SDS	ng/mg	1.45 (1.3)	16.36 (6.87)	104. 94 (57.37)	68.89 (42.97)	104.94 (57.37)
	OD260/280	2.22 (0.44)	1.84 (0.31)	2.22 (0.26)	1.96 (0.07)	2.48 (0.28)
	PCR (%)	791/1 (11), 500/0 (0), 120/4 (44)	791/2 (67), 500/1 (33), 114/3 (100)	114/3 (100)	791/4 (67), 500/4 (67), 120/6 (100)	791/6 (67), 500/6 (67), 120/9 (100)
EDTA + SDS	ng/mg	5.26 (1.92)	6.52 (2.7)	138.18 (38.51)	89.3 (109.59)	3.93 (3.74)
	OD260/280	1.64 (0.42)	1.83 (0.34)	2.19 (0.29)	2.10 (0.11)	2.59 (0.26)
	PCR (%)	791/2 (22), 500/3 (33), 120/3 (33)	791/2 (67), 500/2 (67), 114/2 (67)	114/3 (100)	791/5 (83), 500/3 (50), 120/6 (100)	791/4 (44), 500/6 (67), 120/7 (78)
sum EDTA^c^	ng/mg	4.65 (3.05)	7.5 (5.74)	94.67 (45.98)	51.35 (59.46)	4.89 (4.01)
	PCR (%)	791/10 (24), 500/12 (27), 120/22 (49)	791/10 (67), 500/7 (47), 114/12 (80)	114/15 (100)	791/24 (80), 500/19 (63), 120/29 (97)	791/26 (58), 500/28 (62), 120/31 (69)
Best Protocol + 48 h, 25°C	ng/mg	1.60 (0.40)	11.25 (1.77)	55.00 (7.07)	n.a.	n.a.
	PCR (%)	791/0 (0), 500/5 (56), 120/4 (44)	791/0 (0), 500/2 (67), 114/3 (100)	114/6 (100)		
Best Protocol + 72 h, 25°C	ng/mg	<0.0001	1.33 (0.18)	24.75 (6.72)	n.a.	n.a.
	PCR (%)	791/0 (100), 500/2 (22), 120/2 (22)	791/0 (0), 500/2 (67), 114/3 (100)	114/6 (100)		
Best Protocol + 6 h, 37°C	ng/mg	<0.0001	<0.0001	<0.0001	n.a.	n.a.
	PCR (%)	791/1 (11), 500/2 (22), 120/2 (22)	791/1 (33), 500/3 (100), 114/3 (100)	114/6 (100)		

^a^ pure DNA is indicated by a ration of ~1.8. Lower ratios indicate presence of contaminants (proteins, phenols), higher ratios indicate presence of RNA.

^b^ average DNA yield and amplification success of lysis buffers with CTAB as main component.

^c^ average DNA yield and amplification success of lysis buffers with EDTA as main component.

Within writing supports, palm leaf showed the best results in terms of DNA retrieval and PCR amplification success (up to 138.2 ng/mg and 100% amplification success irrespective of extraction method, see Table 4), followed by papyrus (16.4 ng/mg) and paper (8.7 ng/mg). Although assessment of DNA quantity using fluorescent dyes is a widely used approach, there are some drawbacks in accuracy. Unlike quantification via UV spectrophotometry (such as Nanodrop), fluorometric measurements seem not to be influenced by the presence of protein or RNA contaminants [57], but will underestimate DNA quantity in case of DNA fragmentation [58]. An underestimation of DNA yield in case of fragmented historic samples therefore cannot be excluded.

A negative correlation was observed between DNA yield and PCR amplification success for paper. Using EDTA as main component of the lysis buffer, DNA yield was significantly higher (t test p-value = 0.003, Table 3), but overall PCR performance dropped (t test p-values = 0). While no difference in PCR performance was detected for the shorter fragment (i.e. 120 bp), longer fragments were more often successfully amplified using CTAB as main lysis buffer component for paper (44% compared to 27% for the 500 bp fragment and 40% compared to 22% for the 791 bp fragment, see Table 4). With the exception of papyrus, amplification performance in general was better when CTAB was used as main component of the lysis buffer (t test p-values< 0.045), maybe because CTAB is more efficient in removing polyphenols and polysaccharides which are known to inhibit PCR amplification [59]. Spiking PCR of a subset of EDTA lysed samples did not indicate inhibition. Also diluting DNA template did not enhance PCR success. EDTA has an inhibitory effect on PCR performance, as it depletes divalent cations, such as Mg^2+^, necessary for PCR performance. Depending on the concentration of EDTA, the inhibitory effect will occur randomly, though it has also been noted that some amplicons may be more susceptible to inhibition [60].

**Fig 2:**
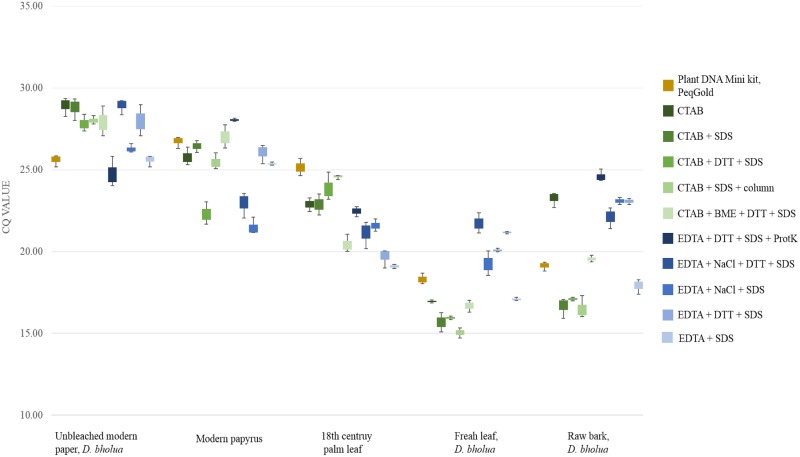
C_q_ values obtained for the rbcl region from different extraction methods. Each extract was tested in triplicate for the plant specific rbcl region.

Sufficient amounts of amplifiable plant DNA were measured irrespective of the applied extraction method (indicated by C_q_ values below 29, Fig 2). C_q_ values were lower for unprocessed material than for writing supports.

DNA purity was assessed using the absorbance ratio A260/A280. While a ratio of ~1.8 indicates pure DNA and higher ratios are indicative of the presence of RNA, lower ratios point to the presence of impurities by proteins or phenol. Sample purity was comparable between the Plant DNA kit and CTAB based lysis buffer (1.5 to 1.9 and 1.4 to 2.1, respectively), while EDTA based lysis buffers showed the lowest sample purity (as low as 0.55, Table 4). A comparison of amplification success of extracts with low DNA purity (≤1.65) to amplification success of DNA extracts with high purity (= 1.8 ±0.02) did not indicate a correlation between purity and PCR success, similar to findings in other studies [61].

**Fig 3:**
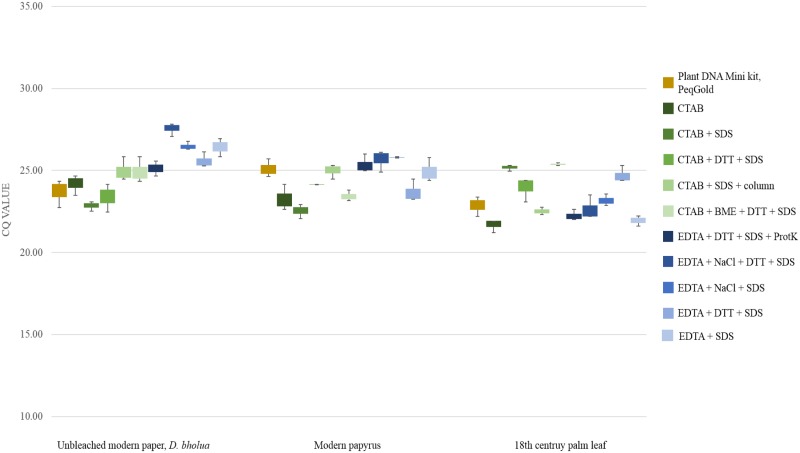
C_q_ values obtained for bacterial 16S rRNA from writing supports. Each extract was tested in triplicate.

Overestimation of DNA retrieval due to the presence DNA contaminations was tested by targeting 123 bp of bacterial 16S rRNA. Real-time qPCR did not indicate a bias in the fluorometric measurements of DNA yields. Rather, bacterial DNA was co-extracted in equally high amounts independent of the extraction method (Fig 3).

Using the best protocol in terms of DNA retrieval for each respective plant based writing support and changing lysis time (6 h at 37 °C, and 48 h and 72 h, each at 25 °C) had a negative effect on DNA yield (t test p-values< 0.05) as well as PCR amplification success after 72 h incubation (t test p-value = 0.010). One possible explanation is DNA degradation due to components of the lysis buffer.

### Minimally destructive and non-invasive sampling of endogenous DNA

The amount of sample material used for DNA extraction was reduced in the course of the experimental set up to 1 mg. In case of papyrus and the historic palm leaf, lower material input correlated with higher DNA yields (t test p-values< 0.05, Table 5). The volume of lysis buffer was probably too low for the lysis of higher material amounts (i.e. 10 mg) and might need to be adjusted in case more material is used for extraction. For the palm leaf manuscript, this corresponded to approximately 2 x 2 mm and produced up to 158 (±38.2) ng DNA. For papyrus, 1 mg corresponded to *c*. 2 x 3 mm and produced up to 21.75 (±2.5) ng DNA. For paper, 1 mg of material corresponded to approximately to 4 x 5 mm and yielded up to 11.1 (±2.4) ng.

**Table 5:** T test results on the influence of material input on DNA output. p-values< 0.05 are highlighted. n = sample size.

Test	DF	T	p-value
Differences in DNA yield with different material input^a^:			
Fresh alcohol preserved leaves: min, max (n = 2)	10	0.479	0.642
Raw bark: min, max (n = 3)	10	1.471	0.172
Modern unbleached paper: min, max (n = 3)	10	-0.544	0.599
**Papyrus: min, max (n = 1)**	10	**4.352**	**0.001**
**Palm leaf: min, max (n = 1)**	10	**2.471**	**0.033**

^a^ minimum material input: 10 mg for the PeqGOLD Plant DNA kit and 1 mg for custom buffers, maximum material input: 30 mg for the PeqGOLD Plant DNA kit and 10 mg for custom buffers.

Although non-destructive sampling is the method of choice for studying cultural heritage and small specimens, invasive sampling is preferable due to higher DNA recovery and a lower risk of extracting contaminant DNA. Especially writing supports will be exposed to high levels of human contamination introduced during manufacturing and usage, and may additionally contain other organic sources involved in the manufacturing of sheets (e.g. glues, sizes).

DNA sampling using eraser proved to be a valuable method for the analysis of parchment [37] and herbaria [31]. Although no DNA was measured after DNA extraction, eraser sampling produced PCR amplification products and authentic sequences for the palm leaf manuscript, while amplification failed for papyrus. In case of paper, eraser sampling proved not to be non-invasive. Asian paper is of filigree texture and rubbing resulted in the damage of the surface.

Using positively charged nylon membranes, low amounts of DNA were recovered from all plant derived writing supports as long as membranes were moistened prior to sampling (up to 0.18 ng/μl for paper, 0.06 ng/μl for papyrus, and 0.4 ng/μl for the historic palm leaf manuscript, Table 6).

**Table 6:** Results of non-invasive DNA sampling of writing supports. n = sample size. Each sampling method was tested twice on each material. PCR was conducted in triplicate for each sample. PCR success is given as the number of PCR products (visualized by gel electrophoresis) for different amplicon lengths (791 bp, 500 bp, and 114/ 120bp). n.a. = not analyzed.

Method		unbleached, modern paper (n = 3)	modern papyrus (n = 1)	18^th^ century palm leaf (n = 1)
Eraser DNA	ng/μl	n.a.	<0.0001	<0.0001
	PCR (%)		791/0 (0), 500/0 (0), 114/0 (0)	114/6 (100)
Nylon DNA moist	ng/μl	<0.0001–0.18	<0.0001–0.06	<0.0001–0.4
	PCR (%)	791/1 (11), 500/3 (33), 120/4 (44)	791/2 (67), 500/2 (67), 114/2 (67)	114/6 (100)
Nylon DNA dry	ng/μl	<0.0001	<0.0001	<0.0001
	PCR (%)	791/0 (0), 500/0 (0), 120/0 (0)	791/0 (0), 500/0 (0), 114/0 (0)	114/0 (0)

**Fig 4:**
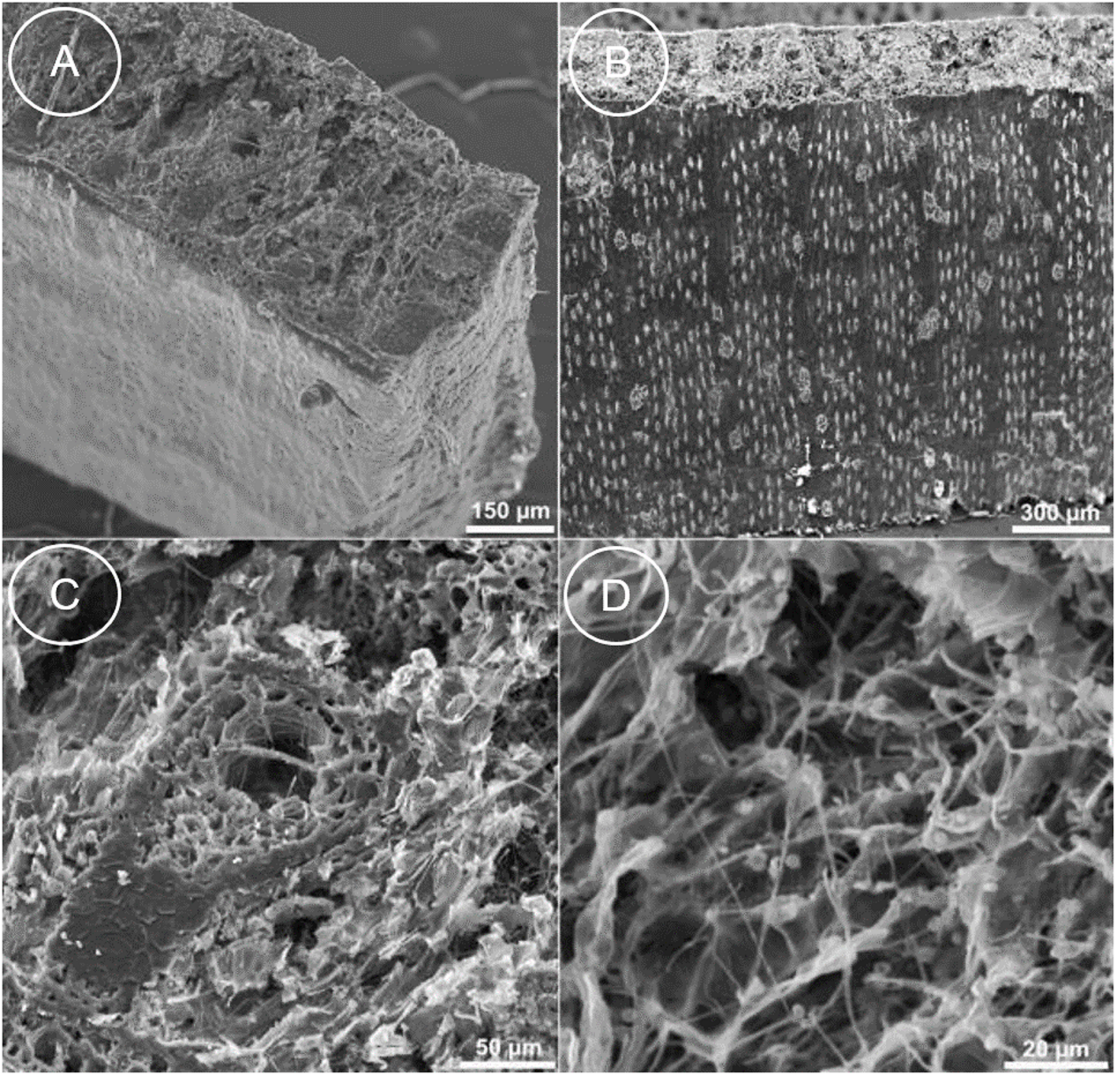
SEM images from the lamina of a palm leaf. (A) Overview of a small leaf sample. (B) The transverse cut direction (above) and the lower leaf surface show the typical structure of a monocotyledonous leaf with parallel lines of stomata that are responsible for gas exchange. (C) A veinlet in the middle of a transverse section shows the xylem and the phloem surrounded by the sclerenchyma sheath and ground tissue. (D) Fungal hyphae occur within the various cell types of the leaf.

Blank controls introduced during sampling (i.e. nylon membranes), DNA extraction and PCR amplification did not indicate contamination. Binding membranes have been so far applied for bacterial and fungal sampling from various surfaces [38–40]. The risk of sampling microbial DNA from the palm leaf manuscript was valid as SEM analyses showed fungal infestation (Fig 4D). Anyway, all sequences retrieved via non-destructive sampling from this sample matched the identified species (see below). For the papyrus sheet, four endogenous sequences out of five were retrieved using nylon sampling, while one contaminant sequence belonged to muskmelon (*Cucumis melo*). The origin of this contamination is unknown. Muskmelon has not been analyzed in the laboratory facilities, therefore contamination during DNA sampling and analysis seems unlikely. For the paper sample made of *Broussonetia papyrifera*, sequences of *Aloe vera* were also retrieved. Aloe was used as sizing agent during paper production and sequences of aloe were also recovered using destructive DNA sampling.

#### Biological origin of the historic palm leaf manuscript

**Fig 5:**
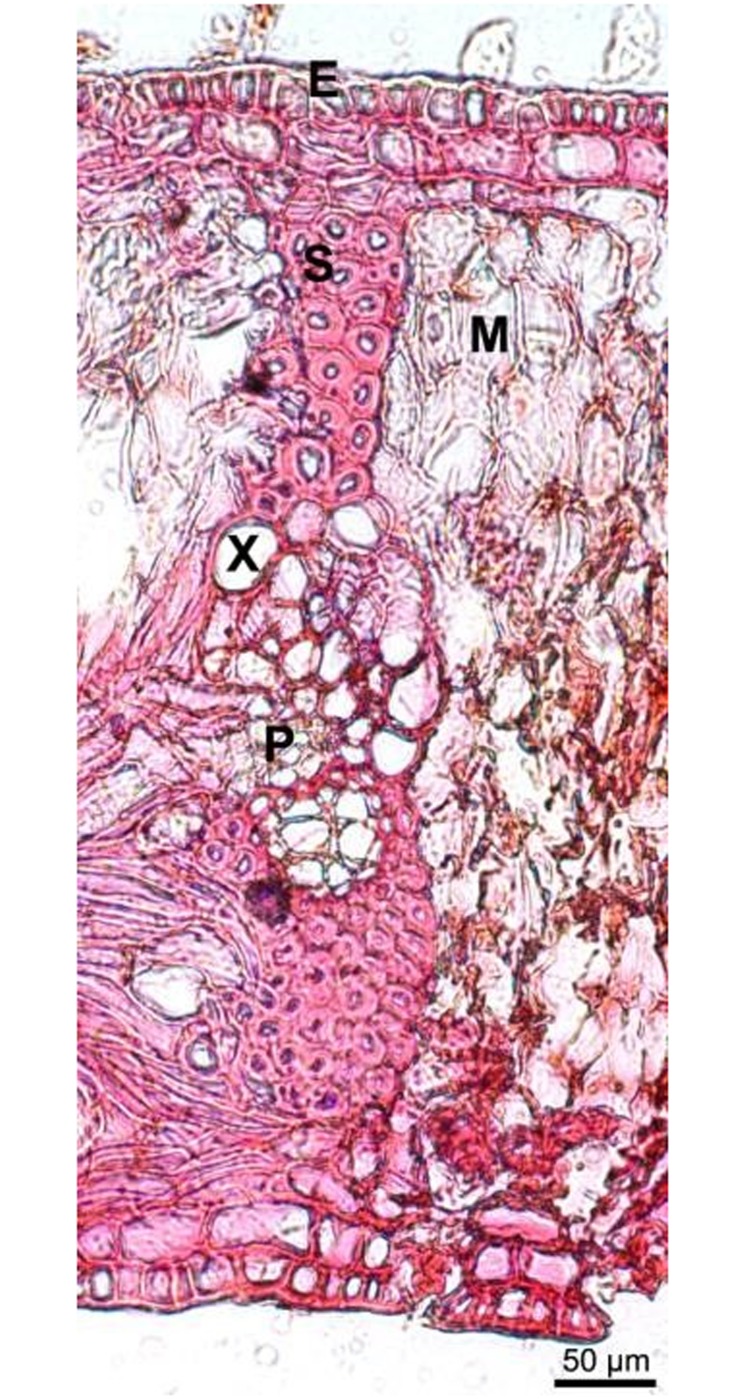
Light microscopic image of a transverse section of a historic manuscript sheet of palm leaf (*Borassus flabellifer*). The dermal system comprises the epidermis (E). The chlorenchymatous ground tissue consists of mesophyll (M) while the vascular system is represented by xylem (X), which is responsible for water flow, and phloem (P), required for assimilate transport. These vascular tissues are stabilized by a sclerenchyma sheath (S).

In case of the palm leaf manuscript, species was identified also using light microscopy and SEM. Light microscopy yielded a complete overview of the leaf from the historic manuscript (Fig 5), in agreement with the structure of a palm leaf taken from a herbarium. Species-specific features within the leaf tissues and on the leaf surface were used to determine the affiliation to palmyra palm (*Borassus flabellifer*). Results were confirmed using SEM (Fig 5). Palmyra palm is one of two major plant species (the other being the talipot palm, *Corypha umbraculifera*) used for the production of palm leaf manuscripts in South Asia.

#### DNA analysis for the identification of paper sources

While microscopic analyses will be sufficient for the identification of plants used for producing writing supports in many cases, some plant fibers cannot be distinguished easily. For example the fibers of *Daphne* and *Edgeworthia*, two plants used for paper production in the Himalayan region [45]. Comparing 748 bp of the chloroplast rbcl (RuBisCo, ribulose-1,5-bisphoshate carboxylase/oxygenase) region of *D*. *bholua* and *E*. *gardneri* with *D. kiusiana* [62] and *E. chrysantha* [63], two other species of *Daphne* and *Edgeworthia*, 12–13 variable positions were found and only one variable position within each genus. We conclude that DNA analyses can be applied to distinguish between paper sheets made of the respective plants.

## Conclusions

Our results indicate, that sufficient DNA can be retrieved from a variety of plant derived manuscripts. Especially the palm leaf manuscript analyzed here showed a good molecular preservation. For paper, it appears that both loss of DNA material as well as some degree of heat induced fragmentation occur during the production of paper. Given that modern paper manufacturing is more detrimental due to the usage of industrial chemicals (i.e. usage of chemicals such as potassium carbonate instead of plant ash as alkaline additive and usage of chemical bleach instead of sun or snow bleaching) our results are promising for future studies on historic paper specimen. Paper products exist in abundance and have been used for purposes other than writing, such as armor, wrapping, and money. Lastly, we show that alternative sampling methods can be applied for analyzing writing supports. Positively charged nylon membranes were successfully applied for nondestructively sample endogenous DNA from delicate material.

## Materials and methods

### Material

Sample material for this study was collected from several plant species used for the production of Asian paper and is listed in Table 7. Modern paper sheets were produced at local workshops. A papyrus sheet was freshly prepared for analysis. Palm leaf of unknown biological origin was directly removed from an 18^th^ century manuscript from a private collection.

**Table 7:** Overview of the samples and DNA methods applied.

Species	Material	Age	DNA analysis method				GenBank accession number
			Extraction comparison	Effect of paper production	Non-invasive sampling	Reference sequence compilation	
*Daphne bholua*	Bark, raw	modern	x			x	MG833726
*Daphne bholua*	Leaf, alcohol preserved	modern	x			x	
*Daphne bholua*	Paper, unbleached	modern	x		x		
*Edgeworthia gardneri*	Bark, raw	modern	x			x	MG833727
*Edgeworthia gardneri*	Leaf, alcohol preserved	modern	x			x	
*Edgeworthia gardneri*	Paper, unbleached	modern	x		x		
*Broussonetia papyrifera*	Bark, raw	modern	x	x			
*Broussonetia papyrifera*	Bark, cooked	modern		x			
*Broussonetia papyrifera*	Paper, unbleached	modern	x	x	x		
*Broussonetia papyrifera*	Paper, bleached	modern		x			
*Broussonetia papyrifera*	Paper, unbleached	early 20^th^ century	(x)^a^	(x)^a^			
*Cyperus sp*.	Papyrus sheet	modern	x		x		
*Borassus flabellifer*	Palm leaf sheet	18^th^ century	x		x		

^a^ Due to limited material, only two extractions were compared (PeqGOLD Plant DNA Mini kit and the EDTA + NaCl + SDS protocol).

All samples were stored at room temperature until further processing.

### Methods

#### Invasive sampling and DNA extraction

Sample preparation and DNA analyses took place at the laboratories of the Hamburg School of Food Science, University of Hamburg.

Prior to DNA extraction, 1 to 30 mg of material was removed using sterile scissors and forceps and cut into small pieces if necessary. In order to test the best extraction method for each respective plant material eleven lysis buffers were compared. Of these, five lysis buffers included cetyl trimethylammonium bromide (CTAB) as main agent, which is commonly used for plant DNA extraction; five buffers included ethylendiaminetetraacetic acid (EDTA) as main component. The method that produced the best result in terms of DNA retrieval for plant derived writing supports was repeated with varying incubation times (48 h and 72 h, each at 25 °C, and 6 h at 37 °C). Lysis buffers used for comparison are given below.

Examination of the influence of paper production on DNA preservation was performed on all sources of *Broussonetia papyrifera* (raw and cooked bark, modern bleached an unbleached paper, historic unbleached paper) with the extraction methods given below. The only exception was the historic paper manuscript. Due to limited material only two lysis buffers were tested on this sample (PeqGOLD Plant DNA Mini kit and EDTA + NaCl + SDS).

PeqGOLD Plant DNA Mini kit modified: 10 to 30 mg of sample material was extracted using the PeqGOLD Plant DNA Mini kit (Peqlab/VWR, Darmstadt Germany) following the manufacturer’s instruction except for an extended incubation time in 500 μl of Lysis Buffer PL1 and 15 μl RNase A for 24 h at 55 °C. DNA material was eluted in 80 μl in two steps of centrifugation after 5 minutes of incubation at 37 °C.

CTAB (modified after [64]): 1 to 10 mg of sample material was incubated in 500 μl of lysis buffer (2% (w/v) CTAB, 100 mM Tris-HCL pH 8.0, 20 mM EDTA pH 8.0, 1.4 M NaCl, 5 μl/ml β-mercaptoethanol, 40 mg/ml PVP) followed by chloroform extraction and isopropanol precipitation. Differences to [64] were an extended incubation time for 24 h at 55 °C in constant agitation and a second extraction with one volume of chloroform. Samples were resuspended in 50 μl of ultrapure water.

CTAB + SDS (modified after [65]): 1 to 10 mg of material was soaked in 600 μl lysis buffer (2% (w/v) CTAB, 100 mM Tris-HCL pH 8.0, 20 mM EDTA pH 8.0, 1.4 M NaCl, 20 mg/ml PVP) and 5 μl of β-mercaptoethanol. After 1 h incubation at 55 °C, SDS was added to each sample to a final concentration of 0.3%. Differences to [65] were less amount of plant material, an extended incubation time in lysis buffer for 24 h at 55 °C in a thermomixer, and two rounds of chloroform extraction. DNA was precipitated in 0.02 volumes of 5 M NaCl and 0.54 volumes of isopropanol followed by two rounds of washing in 70% ethanol. Samples were resuspended in 50 μl of ultrapure water.

CTAB + DTT + SDS: 660 μl of lysis buffer (2% (w/v) CTAB, 100 mM Tris-HCL pH 8.0, 20 mM EDTA pH 8.0, 1.4 M NaCl, 50 mM DTT, 40 mg/ml PVP, 1% SDS) was added to 1 to 10 mg of sample material and incubated for 24 h at 55 °C. Samples were purified twice with one volume of chloroform followed by precipitation as outlined above.

CTAB + silica column: 1 to 10 mg of material was dissolved as described in the CTAB+ DTT+ SDS protocol. After 24 h incubation at 55 °C, samples were centrifuged for 10 minutes at 15.000 x *g*. The supernatant was transferred to microfilters provided in the PeqGOLD Plant DNA Mini Kit and samples were processed according to manufacturer’s instructions. Samples were eluted in two steps of centrifugation after 5 minutes of incubation at 37 °C in a total of 60 μl of elution buffer.

CTAB + BME + DTT + SDS: 660 μl of lysis buffer (2% (w/v) CTAB, 100 mM Tris-HCL pH 8.0, 20 mM EDTA pH 8.0, 1.4 M NaCl, 40 mM DTT, 5 μl/ml β-mercaptoethanol, 40 mg/ml PVP, 1% SDS) was added to 1 to 10 mg of sample material. After 24 h at 55 °C in a heating block, chloroform extraction and precipitation with isopropanol and NaCl were performed as described above.

EDTA + DTT + Proteinase K + SDS: 1 to 10 mg sample material was digested in 650 μl lysis buffer (0.45 M EDTA pH 8.0, 50 mM DTT, 0.25 mg/ml Proteinase K, 1% SDS) for 24 h at 37°C in constant agitation followed by chloroform extraction and precipitation as detailed above.

EDTA + NaCl + DTT+ SDS: 1 to 10 mg sample material was digested in 650 μl lysis buffer (0.43 M EDTA pH 8.0, 0.25 M NaCl, 50 mM DTT, 1% SDS) for 24 h at 37 °C in constant agitation followed by chloroform extraction and precipitation as described above.

EDTA + DTT + SDS: 1 to 10 mg sample material was digested in 650 μl lysis buffer (0.44 M EDTA pH 8.0, 50 mM DTT, 2% SDS). After 24 h at 37 °C in constant agitation, a chloroform extraction was performed and samples were precipitation with isopropanol as described above.

EDTA + NaCl + SDS: 1 to 10 mg sample material was digested in 650 μl lysis buffer (0.43 M EDTA pH 8.0, 0.25 M NaCl, 2% SDS) for 24 h at 37 °C in constant agitation followed by chloroform extraction and precipitation as outlined above.

EDTA + SDS: 1 to 10 mg sample material was digested in 650 μl lysis buffer (0.45 M EDTA pH 8.0, 2% SDS) for 24 h at 37 °C in constant agitation followed by chloroform extraction and precipitation with isopropanol as described above.

#### Non-destructive sampling and DNA extraction

Collection of eraser crumbs (*c*. 15 mg; Mars^®^plastic, Staedtler) was performed as described in [2] with the exception, that eraser crumbs were not collected on paper sheets but were transferred directly from the sampling material into 1.5 ml tubes.

DNA sampling via positively charged nylon membranes (Nytran^®^SuPerCharge, Whatman, GE Healthcare) was performed once with dry membranes and once with membranes moistened with 0.5 M EDTA (pH 8.0). Membranes were cut into pieces (*c*. 1 x 2 cm), pressed for 30 s on the material and transferred to 2 ml tubes containing lysis buffer.

Eraser crumbs and nylon membranes were incubated for 3h at 55 °C in a lysis buffer containing 0.67 M EDTA (pH 8.0) and 1% SDS and were extracted using one volume of chloroform and isopropanol precipitation as outlined above.

#### DNA quantification and fragment size determination

DNA amount was measured using a Quantus^™^ Fluorometer and QuantiFluor ds DNA kit (Promega, Mannheim, Germany). Real-time PCR was used to assess the amount of DNA in each of the eleven extraction methods and was performed in triplicate for each writing support, as well as bark and leaf samples from *D*. *bholua*. Presence of microbial DNA was tested for each of the eleven extraction methods in triplicate for each writing support (historic palm leaf manuscript, modern papyrus sheet and unbleached paper made of *D*. *bholua*). Purity of DNA extracts (OD. 260/280) was measured using a Nanodrop ND-1000 spectral photometer (Thermo Fisher Scientific^™^). PCR amplification success was determined by gel electrophoresis. Fragment sizes were measured on the Bioanalyzer 2100 (Agilent) using the high sensitivity kit.

#### PCR amplification and spiking PCR

**Fig 6:**
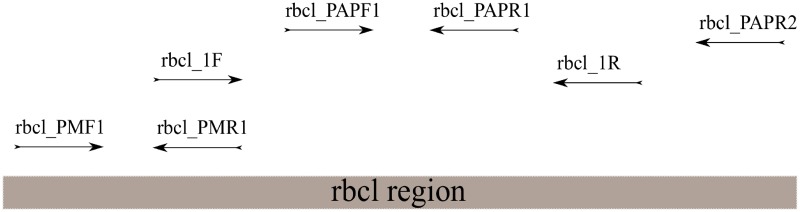
Scheme of primer annealing positions.

Primers were designed using Primer-BLAST [66] and reference sequences for rbcl (RuBisCo, ribulose-1,5-bisphoshate carboxylase/oxygenase) of various plants deposited in GenBank (*Broussonetia papyrifera* [access. no. AF500347], *Daphne mezerum* [access. nos. AF022132, AJ297233], *Daphne laureola* [access. no. HM849946], *Edgeworthia chrysantha* [access. nos. AJ297920, KP088576][63, 67–70], *Borassus flabellifer* [access. nos. KP901247, AY012469], *Corypha umbraculifera* [access. no. AJ404761], *Corypha taliera* [access. no. AJ404762], *Corypha utan* [access. no. AY012466], *Cyperus papyrus* [access. no. Y12966], *Cyperus involucratus* [access. no. Y12967], *Cyperus alternifolius* [access. no. HQ182424]) [63, 67–75]. For the palm leaf manuscript, whose biological origin was unknown prior to analysis, sequences of two palm species used for manuscript production (*Borassus* and *Corypha*) was compiled. For *Daphne bholua* and *Edgeworthia gardneri* no reference sequences were available. In order to generate primers, sequences of related species (*D*. *laureola*, *D*. *mezerum*, and *E*. *chrysantha*) were taken. To account for polymorphic positions at binding sites, primer sequences included ambiguous bases [76]. A scheme of the primer design is shown in Fig 6.

Reference sequences of *D*. *bholua* and *E*. *gardneri* spanning 748 bp of the chloroplast rbcl region were compiled using either two overlapping primer sets (rbcl_1F/rbcl_1R and rbcl_PAPF1/rbcl_PAPR2), or one primer pair (rbcl_1F/rbcl_PAPR2).

PCR performance of the different extraction methods was tested for modern paper plant samples (all material belonging to either *Daphne*, *Broussonetia*, or *Edgeworthia*) using three different primer sets (rbcl_PAPF1/ rbcl_PAPR1, rbcl_1F/ rbcl_1R, and rbcl_1F/ rbcl_PAPR2), that amplify 120bp, 500bp, and 791bp of the rbcl region. For papyrus, rbcl_PAPF1/rbcl_PAPR1 was substituted by rbcl_PMF1/ rbcl_PMR1. Due to fragmentation, only short amplicons were tested for historic samples. Primers are listed in Table 8.

**Table 8:** Overview of the primer sequences and amplicon lengths.

Forward primer name:	Reverse primer name:	Amplicon length (without primers)
sequence (5‘ to 3‘)	sequence (5‘ to 3‘)	
PCR performance of modern samples, compilation of reference sequences of *D*. *bholua* and *E*. *gardneri*:		
rbcl_1F: ACTGATATCTTGGCAGCRTTYCG	rbcl_1R: WCGYGGTGGACTTGATTTTAC	500 (456)
rbcl_1F	rbcl_PAPR2: CTTCACATCCAYCGYGCAAT	791 (748)
Compilation of reference sequences of *D*. *bholua* and *E*. *gardneri*		
rbcl_PAPF1: GTKGGTAATGTATTTGGRTTY	rbcl_PAPR2	530 (489)
PCR performance of papyrus and historic palm leaf, real-time qPCR measurement:		
rbcl_PMF1: CCACAAACAGARACTAAAGC	rbcl_PMR1: ACTGATATCTTGGCAGCATTC	114 (73)
PCR performance of modern paper plant samples and historic paper, real-time qPCR measurement:		
rbcl_PAPF1	rbcl_PAPR1: GGYATCCAAGTTGARAGAGAT	120 (78)

For modern material PCR was set up with 1.2X DreamTaq Buffer (Thermo Fisher Scientific, containing 2.4 mM MgCl_2_), 1 U DreamTaq Polymerase (Thermo Fisher Scientific), 0.4 μg/μl BSA, 0.2 mM dNTP mix (each), 0.1–0.4 μM each primer, and 0.5 to 3 μl of DNA template in a final volume of 20 μl. Initial denaturation for 2 min at 94 °C was followed by 33 cycles of 30 s at each 94 °C, 56 °C and 72 °C, and a final elongation at 72 °C for 10 min.

For historic material PCR reaction was carried out in a final volume of 20 μl containing 1.2x PCR Gold Buffer (Applied Biosystems, Thermo Fisher Scientific), 3 mM MgCl_2_ (Applied Biosystems, Thermo Fisher Scientific), 2.5 U AmpliTaq Gold (Applied Biosystems, Thermo Fisher Scientific), 0.4 μg/μl BSA, 0.2 mM dNTP mix (each), 0.2 μM of each primer, and 1 to 3 μl template DNA. Cycling conditions were 6 min at 94 °C, followed by 39–50 cycles of 40 s at each 94 °C, 56 °C, 72 °C, and a final elongation at 72 °C for 5 min.

Spiking PCR was performed in a final volume of 20 μl by adding 1 μl of spike control to 1.2X DreamTaq Buffer (including 2.4 mM MgCl2), 1 U DreamTaq Polymerase, 0.4 μg/μl BSA, 0.2 mM dNTP mix, 0.1–0.4 μM each primer, and 5 μl of template DNA. Thermal conditions were 2 min at 94 °C, followed by 33 cycles of 30 s at each 94 °C, 56 °C and 72 °C, and a final elongation at 72 °C for 10 min.

Real-time qPCR was set up in a final volume of 20 μl using SYBR^®^ Green I (Sigma Aldrich), 1.2x Dream Taq Buffer (including 2.4 mM MgCl_2_), 1.25 U DreamTaq Polymerase, 0.4 μg/ml BSA, 0.2 mM dNTP mix (each), 0.2 μM of each primer, and 2 μl of template DNA. Primers rbcl PMF1/ R1 were used for the historic palm leaf and papyrus. For material made of *Broussonetia*, *Daphne* or *Edgeworthia* primer pair rbcl_PAPF1/R1 was used. Cycling conditions were 94 °C for 2 min followed by 40 cycles for each 30 s at 94 °C, 56 °C and 72 °C. Primer pair Bact1369F (CGGTGAATACGTTCYCGG) and Prok1492R (GGWTACCTTGTTACGACTT) was chosen to amplify 123 bp of bacterial rRNA [77] with the same conditions as described above A melting curve analysis was performed to monitor for non-specific amplicons.

#### Sequencing and sequence analysis

Prior to sequencing PCR products were purified by enzymatic digestion with 2 U Exonuclease I (Thermo Fisher Scientific) and 0.3 U FastAP Thermosensitive Alkaline Phosphatase (Thermo Fisher Scientific) according to manufacturer’s protocol. Sanger sequencing was carried out externally by GATC Biotech AG. Sequence data were analyzed using MEGA (v.7.0.21) [78].

#### Authentication

DNA analysis was performed in a facility with no prior exposure to paper, palm, or papyrus material. DNA extraction, amplification and post-PCR processing were carried out in separate laboratories in order to prevent carry-over contamination. Workspace and laboratory equipment were wiped with soap and ethanol on a regular basis. PCR was set up in a laminar flow workstation with UVC device. To monitor possible contaminations of the reagents blank controls were processed in parallel during extraction and PCR. For non-destructive sampling eraser material and nylon membranes with no exposure to DNA specimens were co-analyzed.

The authenticity of reference sequences from *D*. *bholua* and *E*. *gardneri* was provided by DNA extraction and PCR amplification of different sample sources (leaf and bark). Sequences were authenticated by at least two extractions and at least three independent PCR amplifications.

The authenticity of sequence results generated with non-invasive DNA sampling techniques was tested by independent DNA sampling, extraction and PCR set up.

#### Data quantification & statistical analyses

Each of the eleven extraction methods was performed with three different sample weight inputs resulting in six extractions per material per method. Details are given in Table 9.

**Table 9:** Scheme of DNA extraction comparisons and PCR amplification.

Material: sample	mg sample input PeqGold Plant DNA Mini kit^a^	mg sample input custom made lysis buffers	n extraction	n PCR amplification
Fresh leaf:			2x/ sample input = 6x/ material/ method	3x/ sample = 6x/ method/ primers set
*D*. *bholua*	10, 20, 30	1, 5, 10		
*E*. *gardneri*	10, 20, 30	1, 5, 10		
Raw bark:			2x/ sample input = 6x/ material/ method	3x/ sample = 9x/ method/ primer set
*D*. *bholua*	10, 20	5, 10		
*E*. *gardneri*	20, 30	1, 5		
*B*. *papyrifera*	10, 30	1, 10		
Modern paper, unbleached:			2x/ sample input = 6x/ material/ method	3x/ sample = 9x/ method/ primer set
*D*. *bholua*	10, 20	1, 10		
*E*. *gardneri*	20, 30	5, 10		
*B*. *papyrifera*	10, 30	1, 5		
Modern papyrus	10, 20, 30	1, 5, 10	2x/ sample input = 6x/ sample/ method	3x/ sample = 3x/ method/ primer set
18^th^ century palm leaf	10, 20, 30	1, 5, 10	2x/ sample input = 6x/ sample/ method	3x/ sample = 3x/ method/ primer set

^a^ 10 to 50 mg of dry material are recommended by the manufacturer.

Influence of incubation time on DNA yield was tested on all writing supports Non-destructive DNA sampling was performed twice on all modern unbleached paper sheets, papyrus and palm leaf. Influence of paper production on DNA preservation was examined by DNA extraction using different lysis buffers, resulting in eleven DNA extractions for each material except for the historic paper sheet. Due to limited material only two extractions were performed on this sample. Details on experimental set up are listed in Table 10. Standard deviations were calculated using the STEDV function in MS Excel (2017). Paired t tests were calculated using SPSS software 25.

**Table 10:** Scheme of paper production comparisons and PCR amplification.

Material	mg sample input PeqGold Plant DNA Mini kit^a^	mg sample input custom made lysis buffers	n extraction	n PCR amplification
*B*. *papyrifera*: Raw bark	10	1	1x/ sample input = 11 extractions	3x/ extraction = 33 PCR amplifications/ primer set
*B*. *papyrifera*: Cooked bark	30^a^	10^a^	1x/ sample input = 11 extractions	3x/ extraction = 33 PCR amplifications/ primer set
*B*. *papyrifera*: Paper, modern, bleached	10	5	1x/ sample input = 11 extractions	3x/ extraction = 33 PCR amplifications/ primer set
*B*. *papyrifera*: Paper, modern, unbleached	10	5	1x/ sample input = 11 extractions	3x/ extraction = 33 PCR amplifications/ primer set
*B*. *papyrifera*: Paper, early 20^th^ century	10	5	2 extractions (PeqGold Plant DNA Mini kit & EDTA, NaCl + SDS)	3x/ extraction = 6 PCR amplifications

^a^ because this sample was wet, more sample material was tested.

#### Light microscopy

Palm leaf material was sectioned into 5 mm long segments and soaked in pure water for one week. Subsequently the segments were treated with 30% PEG for one week and then embedded in 100% PEG. 20 μm thick sections were cut with a microtome and stained with 1% safranin for light microscopy. Images of palm sections were made using a light microscope (Zeiss Axioscope 40) equipped with a digital camera (Zeiss AxioCam MRc). Structural analysis was performed using ZEN 2012 (Zeiss software blue edition service pack 2).

#### SEM

Small sections of leaf tissue were cut with a razor blade. After drying, the samples were coated by carbon (BIO-RAD SEM Coating System) and examined in a scanning electron microscope (Hitachi S520, Japan).
